# DMSF-YOLO: Cow Behavior Recognition Algorithm Based on Dynamic Mechanism and Multi-Scale Feature Fusion

**DOI:** 10.3390/s25113479

**Published:** 2025-05-31

**Authors:** Changfeng Wu, Jiandong Fang, Xiuling Wang, Yudong Zhao

**Affiliations:** 1 College of Information Engineering, Inner Mongolia University of Technology, Hohhot 010051, China; changfengwu69@gmail.com (C.W.);; 2 Inner Mongolia Key Laboratory of Intelligent Perception and System Engineering, Hohhot 010080, China; 3 Inner Mongolia Synergy Innovation Center of Perception Technology in Intelligent Agriculture and Animal Husbandry, Hohhot 010080, China

**Keywords:** cow, behavior recognition, YOLOv11, dynamic mechanism, multi-scale feature fusion

## Abstract

The behavioral changes of dairy cows directly reflect their health status, and observing the behavioral changes of dairy cows can provide a scientific basis for dairy farms so managers can take timely measures to intervene and effectively prevent diseases. Because of the complex background, multi-scale behavior changes of dairy cows, similar behavior, and difficulty in detecting small targets in the actual dairy farm environment, this study proposes a dairy cow behavior recognition algorithm, DMSF-YOLO, based on dynamic mechanism and multi-scale feature fusion, which can quickly and accurately identify the lying, standing, walking, eating, drinking and mounting behaviors of dairy cows. For the problem in multi-scale behavior changes of dairy cows, a multi-scale convolution module (MSFConv) is designed, and some C3k2 modules of the backbone network and neck network are replaced with MSFConv, which can extract cow behavior information of different scales and perform multi-scale feature fusion. Secondly, the C2BRA multi-scale feature extraction module is designed to replace the C2PSA module, which can dynamically select the important areas according to the two-layer routing attention mechanism to extract feature information at different scales and enhance the multi-scale feature extraction capability of the model, and the same time inhibit the interference of the background information to improve the small target detection capability of the model. Finally, the Dynamic Head detection head is introduced to enhance the model’s scale, spatial location, and perception of different tasks, enhance the capacity to extract similar behavioral features of cows, and improve the model’s performance in detecting cow multi-scale behaviors in complex environments. The proposed DMSF-YOLO algorithm is experimentally validated on a self-constructed cow behavior dataset, and the experimental results show that the DMSF-YOLO model improves the precision (P), recall (R), mAP50, and F1 values by 2.4%, 3%, 1.6%, and 2.7%, respectively, and the FPS value is also high. The model can suppress the interference of background information, dynamically extract multi-scale features, perform feature fusion, distinguish similar behaviors of cows, enhance the capacity to detect small targets, and significantly improve the recognition accuracy and overall performance of the model. This model can satisfy the need to quickly and accurately identify cow behavior in actual dairy farm environments.

## 1. Introduction

With the continuous development of modern agriculture and animal husbandry, the increasing attention to sustainable agriculture and animal welfare has made dairy cow health management increasingly important [1,2]. The identification of cow behavior has become critical as cow behavioral patterns are closely related to cow health status, as well as to the yield and quality of dairy products [3,4,5]. Rapid and accurate identification of cow behavior can help dairy farm managers detect potential health problems of cows in time, and then take effective management measures to improve the overall welfare of cows and the economic benefits of dairy farms.

At present, there are three main methods for cow behavior recognition, namely manual observation, sensor detection, and deep learning-based target detection [6,7,8]. Although manual observation is intuitive and flexible, it is costly, inefficient, and easily interfered with by human factors, resulting in missed detection [9]. Sensor detection is mainly used to obtain the cow’s activity data by attaching sensors (e.g., accelerometers, gyroscopes, etc.) to the cow and then identifying the specific behavior of the cow by analyzing the data. Although this method can achieve high precision and has all-weather detection, it may produce a stress reaction in the cow as well as cause damage to the sensor equipment [10,11]. In contrast, deep learning-based target detection technology has significant advantages, and through an automated monitoring and intelligent analysis system, it can achieve contactless recognition of the behavior of cows and improve the detection efficiency while maintaining a high accuracy rate [12,13].

Nowadays, domestic and foreign researchers mainly adopt the target detection model based on deep learning to extract the behavioral features of cows through image processing technology for cow behavior recognition [14,15]. To systematically analyze the current development status and problems in the field, in this study, during the literature research process, we focus on the cow behavior recognition task with deep learning models, and select relevant papers from mainstream journals to ensure the representativeness and cutting-edge nature of the review. Li et al. proposed an improved RefineMask instance segmentation model aimed at recognizing the eating behavior of cows to solve the problems of low recognition accuracy and high error rates in the recognition of cows’ behavior. The algorithm introduces the convolutional block attention module and the GIoU loss function, and then adds a channel attention module to the neck. Finally, the logic for identifying eating behavior using mask information is designed, and the model can accurately determine the eating behavior of cows. However, this model only recognizes the single behavior of eating, which limits its applicability to the recognition of multiple behaviors of dairy cows in actual scenarios [16]. Wang et al. proposed an E3D algorithm to identify cow behaviors (lying, standing, walking, drinking, and eating), which is used to solve the problems of existing algorithms that have a large number of parameters and are difficult to be deployed at the edge. After introducing the 3D volume and ECA module, the algorithm has a small number of parameters, and the recognition results are accurate and efficient, but the algorithm cannot effectively identify small target cows [17]. Zong et al. proposed a cow multi-scale behavior recognition algorithm based on YOLOv5 to cope with the complex environment and the changes in cow multi-scale behavior in actual dairy farms. The algorithm integrated four Shuffle Attention (SA) modules in the neck network of the YOLOv5 model. At the same time, it combined the C3 module of the deformable convolution (DCNv3) enhancement model, and finally introduced DyHead, the improved model that has a higher accuracy in recognizing cow behaviors. However, the model does not recognize a cow’s mounting behavior and cannot determine a cow’s estrus period [9]. Bloch et al. proposed a CNN-based classification and recognition model for dairy cow eating behavior and used transfer learning to improve the performance of the model. The model has a high recognition accuracy for dairy cow feeding behavior. However, the cow behaviors identified by this method are not diverse enough and lack practical applicability [18]. Yuan et al. used an improved YOLOv8 model to identify calf behavior (walking, standing, lying, eating, and drinking), aimed to solve the problems of cow occlusion and different lighting in the complex environment of actual dairy farms. The model introduced the P2 small target detection layer and reduced the computational complexity of the model through the Lamp pruning method. The results showed that the improved model is more lightweight, more accurate, and more real time, but cannot effectively cope with the multi-scale behavioral changes of dairy cows [19]. Qiao et al. proposed a cow behavior classification model called C3D-ConvLSTM to address the problem that the behavioral actions of cows are similar and difficult to recognize and classify. The method classifies five common cow behaviors, including eating, exploring, grooming, walking, and standing, by using C3D and Convolutional LSTM, and the proposed model can accurately classify cow behaviors. However, this method cannot easily distinguish similar behaviors of cows, and the recognition accuracy of similar behaviors is insufficient [20]. Yu et al. proposed a model called Res-DenseYOLO that can accurately detect the standing, lying, eating, and drinking behaviors of dairy cows. By introducing a dense module (DenseNet), CoordAtt attention mechanism, and SioU loss function in YOLOv5, and adding a small target detection head, the results showed that the model has a significant improvement in cow behavior recognition and can accurately detect cow behaviors in real time. However, the model does not easily reduce the interference of environmental factors in complex environments, which brings challenges to the recognition of dairy cow behaviors [21].

In summary, in the large-scale dairy farm environment, due to the complexity of the background, the multi-scale changes in cow behavior, the diversity of behavior, and the difficulty in detecting small targets, the recognition of cow behavior poses many challenges, which seriously affect the accuracy and reliability of cow behavior recognition [16,17,18,19,20,21]. The aim of this study is to be able to cope with the challenges brought about by the multi-scale behavioral changes of cows in the actual dairy farm environment, enhance the recognition ability of small target cows, effectively suppress the interference of the background, and be able to quickly and accurately identify the different behaviors of cows. In response to the above problems, we propose a cow behavior recognition algorithm, DMSF-YOLO, based on a dynamic mechanism and multi-scale feature fusion, and the contributions of this study are as follows:

- For the model to meet the application in real scenarios, this study uses a combination of camera and surveillance to collect image data of cow behavior, and after labeling the cow behavior categories with Labelimg software, data enhancement is carried out to construct the cow behavior dataset.
- It designs a multi-scale convolution module (MSFConv). This module has four convolution kernels of different sizes, which can extract local and global features from multiple receptive fields in parallel and effectively capture cow behavior characteristics at different scales. Then, the channel partitioning mechanism is combined to realize multi-scale feature extraction, and then a 1 × 1 convolution is used to efficiently fuse features of various scales, which reduces the amount of calculation and enhances the model’s capability to recognize cow multi-scale behaviors.
- It designs the C2BRA module, which combines BiLevelRoutingAttention (BRA) with C2PSA. C2BRA can dynamically filter the key areas and pixels in the multi-scale feature map through dynamic sparse attention and a dynamic routing mechanism, focus on the key features of cow behavior, and enhance the perception and semantic alignment of cow targets of different scales. This module enhances not only the capability of cow multi-scale feature extraction and fusion, but also the capability of small target detection; it also effectively suppresses background interference.
- By replacing the Detect detection head of the original model of YOLOv11n with Dynamic Head (Dynamic Detection Head), it can obtain key local features and dynamically assign feature weights, automatically weigh the features of different layers, enhance the recognition ability of large and small targets, focus on the key parts of the cow, reduce the interference of irrelevant background factors, enhance the ability of similar behavioral feature extraction, and improve the performance of the model for multi-scale target detection.

## 2. Materials and Methods

### 2.1. Experimental Data

#### 2.1.1. Data Sources

**Figure 1:**
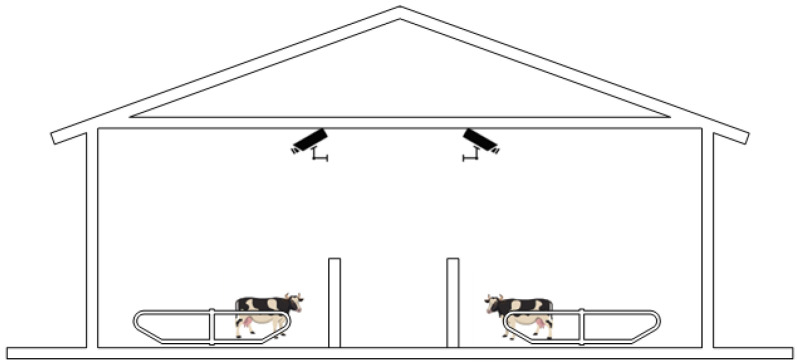
Cowshed camera installation location diagram.

**Figure 2:**
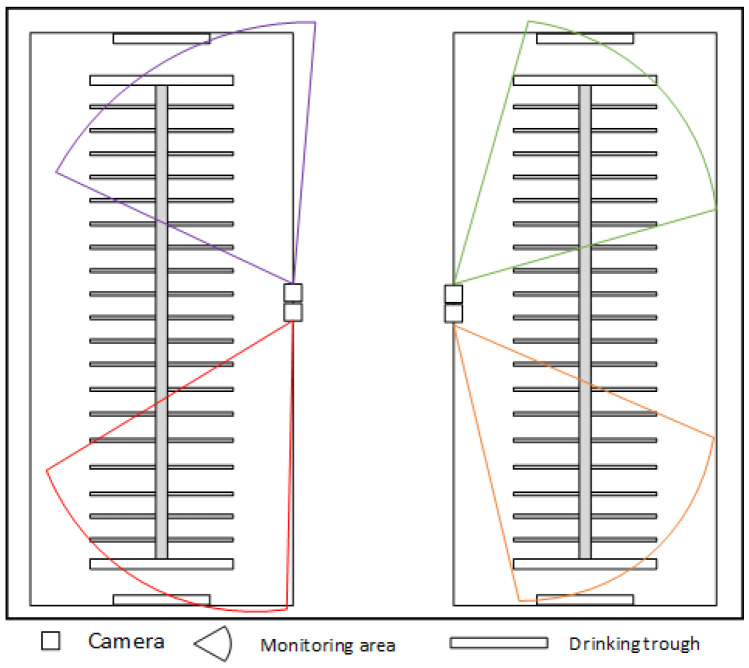
Monitoring coverage diagram.

The experimental data of this study were collected in the field at Mengdelong Dairy Farm and Shengqingyuan Dairy Farm in Hohhot City, Inner Mongolia Autonomous Region, both of which are dairy farms with a stock of about 500 cows. We used a combination of camera and surveillance collection to obtain images of cow behavior. In the actual farm environment where the experimental data were collected, the dairy farms utilized surveillance cameras installed on the beams of the double-sloped barns to monitor the cows’ activity areas, using high-definition cameras with a monitoring distance of 40 m and at a height of about 3 m above the ground. The dairy farms installed multiple surveillance cameras on the barn beams. As shown in Figure 1 and Figure 2, this arrangement can achieve full coverage of the cow activity area, record cow behavior from multiple viewpoints, and better capture the behavioral characteristics of the cows, as well as effectively reduce the lack of data due to obstruction or monitoring blind zones, and ensure that the behavioral image data captured are comprehensive and of high quality.

#### 2.1.2. Dataset

To improve the robustness of the model, so that the model algorithm can be applied to the detection of cow behavior in multi-scene and complex environments, this study constructs a cow behavior dataset by using a combination of camera and frame extraction from the surveillance video to obtain the images of cow behaviors, which include six behaviors: lying, standing, walking, eating, drinking, and mounting. As shown in Table 1, these six behaviors are common in the daily life of dairy cows. Their changes can directly reflect the health status and welfare level of dairy cows. In addition, these six behaviors have clear action patterns and strong identifiability in the video, which is convenient for model training and data annotation, improving the accuracy and practicality of overall detection. Firstly, the robustness of the model can be improved through multi-scene and multi-angle shooting with the camera. Secondly, the video frame extraction technique is used to extract images from the surveillance video, which not only helps to build richer time-series data but also effectively reduces sample bias due to chance factors. Combining data from these two sources not only enhances the representativeness of the dataset but can also depict the behavioral characteristics of cows more comprehensively and improve the adaptability of the algorithm to complex scenarios to achieve higher accuracy and reliability in practical applications.

In this study, 8 cow monitoring videos were selected; the resolution of the monitoring video is 1080P, the number of pixels is 1920 × 1080, and the video frame rate is 25 fps. The video was extracted every 100 frames, and the extracted images were manually selected to remove redundant images to avoid too much similarity between neighboring frames. The images captured by the camera and the images obtained by frame extraction were labeled using the Labelimg software, and different behavioral categories were labeled according to different behavioral characteristics of the cows to establish the cow behavior dataset. To ensure the consistency and accuracy of the dataset, the cow behavior dataset in different scenarios is divided into a training set, validation set, and test set according to a ratio of 7:2:1. The dataset is expanded by using data augmentation, and the new cow behavior dataset after expansion has a total of 4632 images, comprising 3234 images in the training set, 918 images for in validation set, and 480 images in the test set.

**Figure 3:**
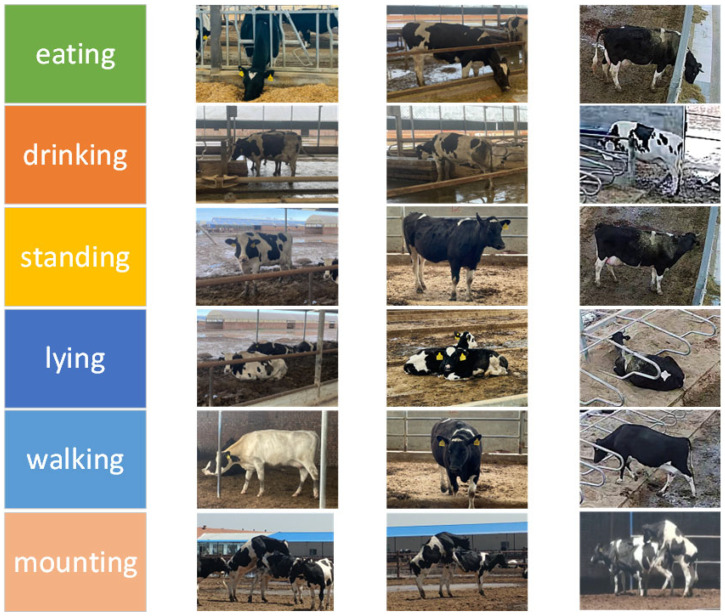
Example of cow behavior images.

The number of labels in the dataset is counted, and a total of 31,824 behavioral labels are labeled according to the analysis of the number of different behavioral categories. Among them, the number of cows lying and standing is the highest, followed by the number of cows walking and eating. The proportion of cows drinking is relatively low, and the proportion of cows mounting is the smallest. The distribution of behavior category labels in the dataset aligns with the actual behavioral patterns of cows in real-world dairy farm environments, thereby reflecting their typical activity profiles [20]. Figure 3 shows an example diagram of the determination of different behavioral categories of cows.

#### 2.1.3. Dataset Analysis

**Figure 4:**
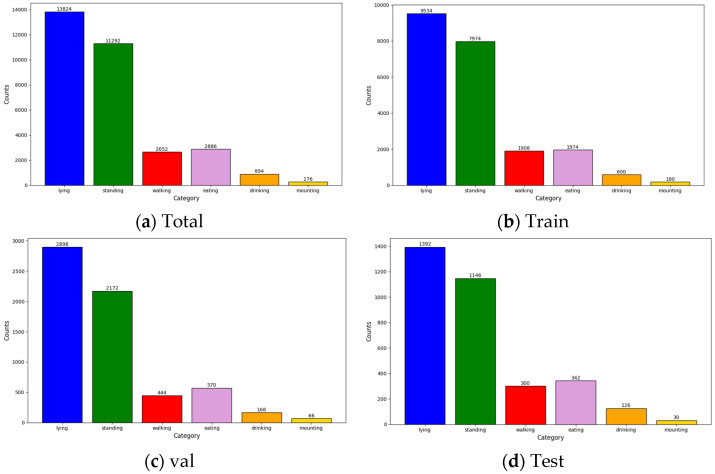
Distribution of labels in the cow behavior dataset.

As shown in Table 2, in this study, a data stratification strategy is adopted in constructing the dataset, and the proportion of each behavioral category in the three subsets of the dataset is ensured to be roughly the same as in the data division process. To further verify the rationality of data division, we conduct a statistical analysis of the distribution of behavior labels in the three subsets of the constructed dataset. As shown in Figure 4, the results show that the trend of the distribution of the category in each subset is basically the same, which avoids the absence or extreme imbalance of a certain category in the validation or testing phase, and ensures the fairness of the training and evaluation of the model and the trustworthiness of the experimental results.

For further study, we visualize several key factors in the cow behavior dataset. The size and number of target boxes can reflect the size of the target and the overall distribution of different targets. Then, the center point position of the target box can not only reflect the spatial distribution characteristics of the target in the image, but also reflect the activity pattern of the cow in a specific area. Finally, the height–width ratio of the target box relative to the entire image can not only reflect the distribution of large and small targets in the dataset, but also reflect the scale of the target.

**Figure 5:**
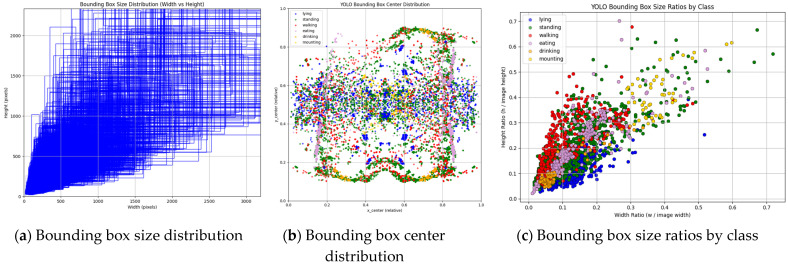
Dataset analysis diagram.

By observing Figure 5, we find that the target boxes are mostly concentrated in smaller sizes, which indicates that there is a large proportion of small targets in the dataset. The targets are mostly concentrated in the center of the image and less at the edge of the image, indicating a reasonable distribution of cow targets. The height–width ratio of the target box relative to the entire image is concentrated in a smaller proportion, which indicates that the dataset not only contains many small targets, but also reflects the multi-scale behavior of cows.

#### 2.1.4. Data Augmentation

**Figure 6:**
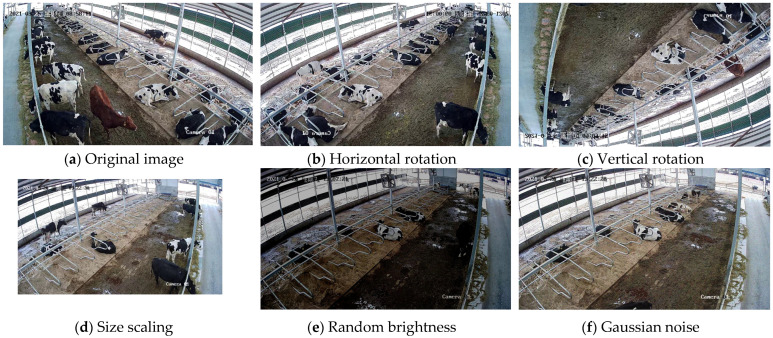
Data enhancement effect diagram.

Considering the problems of large changes in cow scales, rapid changes in cow behavioral poses, variations in lighting, and background clutter commonly found in real dairy farm environments, data enhancement is performed on the established dataset. In this study, the dataset is subjected to five ways of data enhancement: horizontal rotation, vertical rotation, size scaling, random brightness, and Gaussian noise. The effect of data enhancement is shown in Figure 6.

The cow behavior data collected in actual dairy farms are usually diverse and complex, and different angles, lighting, occlusions, etc., may appear in the actual environment. Through data augmentation, the diversity of data can be effectively increased to enrich the background information and multi-scale behavioral information of cows, covering more scenarios of a cow’s life, so that the model can maintain a better performance in the face of different situations, thus improving the robustness and generalization ability of the model [22].

### 2.2. Methods

**Figure 7:**
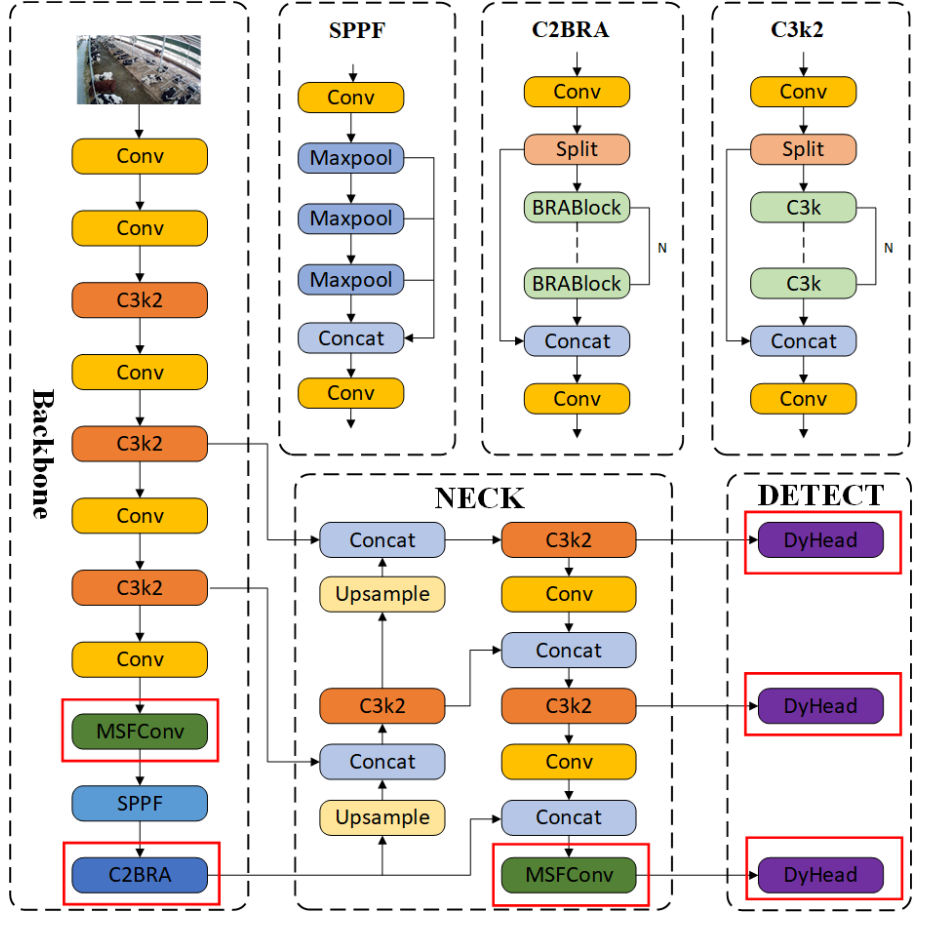
DMSF-YOLO cow behavior recognition network structure diagram.

To accurately identify the behavior of cows, this study selects YOLOv11n as the basic model for cow behavior recognition. To solve the problems encountered in the process of cow behavior recognition, the DMSF-YOLO model is proposed. The DMSF-YOLO cow behavior recognition network architecture is shown in Figure 7, and the improved parts are marked with red rectangles. The specific improvements are as follows:

- MSFConv: The multi-scale convolution module (MSFConv) is designed, utilizing four convolution kernels of different sizes to cover different sensory field regions, and each convolution is responsible for extracting features at various scales to enhance the model’s capacity to recognize multi-scale targets.
- C2BRA: The C2PSA is replaced by C2BRA, i.e., the PSA in C2PSA is replaced by BiLevelRoutingAttention (BRA), which enhances the capacity to extract key features of cow behaviors, reduces the impact of environmental factors, and improves the model’s capacity to perceive cow targets at various scales and detect small targets.
- Dynamic Head: The detection head of the original YOLOv11n model is replaced with the Dynamic Head, a unified target detection head for the attention mechanism, which improves the recognition ability of cow behaviors from the three levels of scale perception, spatial location of spatial perception, and task perception, respectively.

#### 2.2.1. YOLOv11

YOLOv11 has five different sizes of pre-training models, which are N, S, M, L, and X, in ascending order. As the model increases, the detection performance improves, but at the same time, the number of parameters is larger, and the higher the requirement for computational resources.

The YOLOv11 model mainly consists of four parts. The input layer, serving as the initial stage of the entire network, is primarily responsible for receiving and processing image data [23]. The backbone network is responsible for feature extraction, which mainly consists of the convolutional blocks C3k2, SPPF, and C2PSA. The neck network, which mainly consists of the convolutional blocks C3k2, and Upsample, is designed to converge features of different scales, which can realize effective multi-scale feature fusion and deliver it to the head. The detection head employs a dual-head architecture where the model uses two prediction heads during training, one using one-to-many assignment and the other using one-to-one assignment.

#### 2.2.2. MSFConv

**Figure 8:**
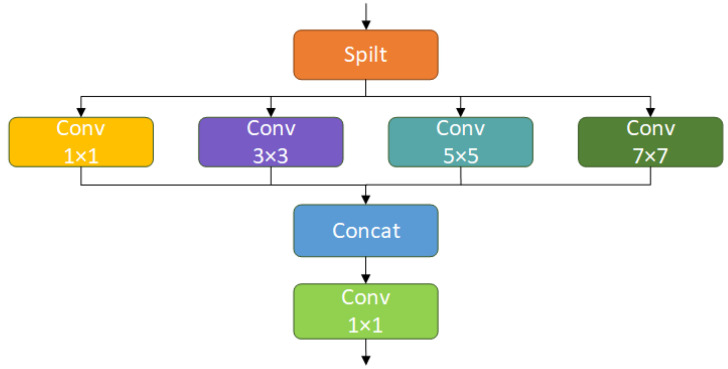
MSFConv network structure diagram.

When monitoring the cow in the actual dairy farm environment, due to the specificity of the installation location of the surveillance camera, different scales of cows will appear at different distances from the camera, which will lead to the scale change of the behavioral characteristics of the cows, and created challenges in detecting the behavior of the cows. To effectively deal with the impact of multi-scale changes and more efficiently integrate cow behavior feature information at different scales, as shown in Figure 8, this study designed a multi-scale convolution module (MSFConv).

The C3k2 module in YOLOv11 uses a fixed-size convolutional kernel (3 × 3) with a single receptive field, which can only extract features at a single scale and cannot easily extract target features at different scales. Different convolution kernels can effectively capture information from different receptive fields in cow behavior images. The MSFConv module designed in this study introduces four convolution kernels of various sizes, 1 × 1, 3 × 3, 5 × 5, and 7 × 7, to form a pyramidal coverage of receptive fields, which enables the parallel extraction of both local and global features and the capture of both local details and global contextual information. Meanwhile, the module adopts a channel division strategy to assign the input features of different scales convolutional branches in groups, which both improves the efficiency of multi-scale feature extraction and reduces the computational overhead. Finally, the fusion is performed by a 1 × 1 convolution, which effectively integrates the multi-scale features while keeping the output dimension consistent and also maintains the simplicity and efficiency of the module.

Replacing part of the C3k2 of the backbone network and neck network in YOLOv11n with MSFConv, the improved model can better extract the information of the cow behavioral feature maps, enhance the perception ability of multi-scale cow behavior targets, realize cross-scale feature fusion, and improve the model’s small-target detection and robustness in complex scenarios.

The main formulation of the multi-scale convolutional enhancement module (MSFConv) is shown below.

(1) $$x_{group}=S(x,g)$$

(2) $$x_{conv}\left[i\right]={Conv}_{k_{i}}\left(x_{group}\left[i\right]\right),i\in [0,\cdots ,g-1]$$

(3) $$x_{merged}=Concat(x_{conv}\left[0\right],\cdots ,x_{conv}[g-1])$$

(4) $$y={Conv}_{1\times 1}(x_{merged})$$

where x is the input tensor, g is the number of groups, ${Conv}_{k_{i}}$ is the i-th convolutional operation, $x_{conv}\left[i\right]$ is the output tensor of the i-th group, $x_{merged}$ is the feature merging, and ${Conv}_{1\times 1}$ is the convolutional fusion.

#### 2.2.3. C2BRA

In actual dairy farms, there are not only multi-scale behavioral changes in cows, but also some small target cows due to the different distances between the target cows and the surveillance camera. In addition, due to the interference of background information around the cows, it is challenging to recognize the multi-category cow behaviors, which are all problems that need to be solved.

C2PSA is a module in YOLOv11n for enhanced feature extraction, combining the CSP (Cross Stage Partial) structure and the PSA (Pyramid Squeeze Attention) attention mechanism. Although C2PSA has multi-scale feature extraction capabilities, it is better suited to local space and channel modeling and has limited feature extraction capabilities for multi-scale changes. In addition, C2PSA is a fixed attention channel and spatial branch, which cannot be dynamically adjusted according to the feature map, and has poor dynamic adaptability. To solve the problems of multi-scale fusion and semantic selectivity in the cow behavior recognition task, this study innovatively replaces the PSA attention mechanism in C2PSA with BiLevel Routing Attention (BRA) to form a new C2BRA module. C2BRA enhances the multi-scale feature fusion capability and regional perception capability through dynamic sparse attention and dynamic routing mechanism, and improves the detection performance of small object targets [24].

C2BRA can dynamically filter key areas and pixels in multi-scale feature maps through double-layer routing (region level + token level), and maintain efficient attention allocation at different scales. The features between different spatial scales are selectively fused with more semantic relevance to focus on key feature areas and reduce background interference. C2BRA can adaptively associate semantic and detailed information at different levels, focus on local key details at the shallow level, capture global contextual semantic information at the deep level, and enhance the semantic discrimination ability of low-level features through cross-level attention to achieve local–global information collaborative modeling.

**Figure 9:**
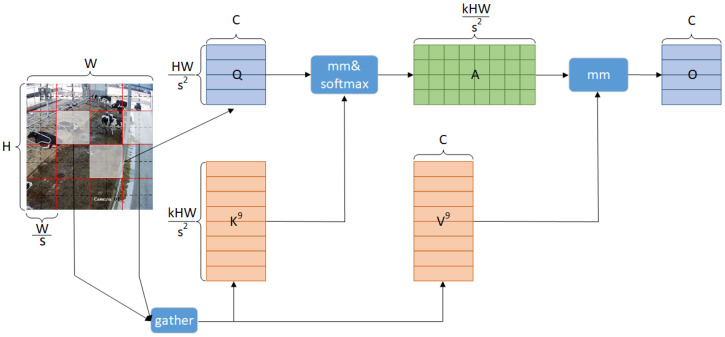
BiLevel Routing Attention (BRA) network architecture diagram.

As shown in Figure 9, this module not only breaks through the channel and spatial separation ideas of traditional PSA in structural design, realizing dynamic allocation of attention, but also optimizes and transforms the multi-scale and context perception requirements of target detection tasks. C2BRA shows significant advantages over C2PSA in multi-scale feature fusion, which not only enhances the detection ability of small target cows, but also effectively suppresses the background interference of dairy farms, focuses on the key areas of target cows, reduces the risk of false detection and missed detection, and helps to enhance the understanding of target edges, details and global context while maintaining model performance.

**Figure 10:**
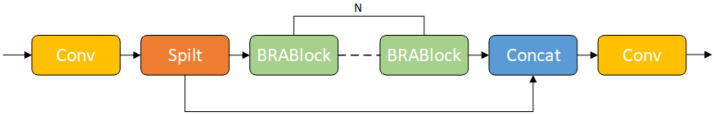
C2BRA network architecture diagram.

As shown in Figure 10, the input feature map $X\epsilon R^{H\times W\times C}$ is first divided into S × S different regions, each containing $\frac{HW}{S^{2}}$ feature vectors, that is X into $X^{\gamma }\epsilon R^{H\times \frac{HW}{S^{2}}\times C}$, and then $Q,K,V\in R^{H\times \frac{HW}{S^{2}}\times C}$ are obtained by linear mapping [24].

(5) $$Q=X^{\gamma }W^{q}$$

(6) $$K=X^{\gamma }W^{k}$$

(7) $$V=X^{\gamma }W^{v}$$

where $W^{q},W^{k},W^{v}$ are the projection weights of Q,K,and V, respectively.

#### 2.2.4. DyHead

When cows approach or move away from the surveillance camera, cows of different scales will appear in the same image. Cow behavior recognition requires the simultaneous detection of small target cows at a distance and large target cows at a close distance. The traditional detection head may be omitted or misdetected due to scale changes and spatial location changes of different behaviors, which requires the detection head to have a better multi-scale perception and spatial perception to deal with the relationship between multi-scale changes and the different spatial location changes of cows. When identifying cow behaviors, the identification of different behaviors depends on the relevant local features, which requires different task perception abilities of the detection head.

To solve the above problem, the DyHead detection head is introduced to coherently combine multiple self-attention mechanisms from within the feature level of scale awareness, the spatial location of spatial awareness, and the output channel of task awareness, respectively, which significantly improves the target detection head’s representation ability [25,26]. DyHead introduces scale-aware attention (scale-attention), which dynamically fuses features for cows with different scale behaviors according to the semantic importance of different scales. Spatial-aware attention is used to process the cow’s behavioral features in the spatial dimension and focuses on the discriminative region between the spatial location and the feature level. Then, the task-aware attention mechanism is used to distinguish different types of behavior recognition tasks, enhance the ability to extract similar cow behavior features, and improve the accuracy of cow behavior recognition.

**Figure 11:**
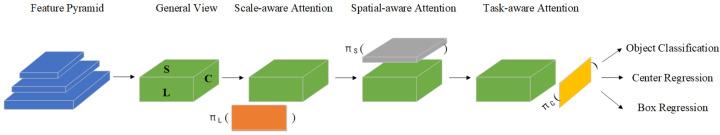
DyHead network architecture diagram.

**Figure 12:**
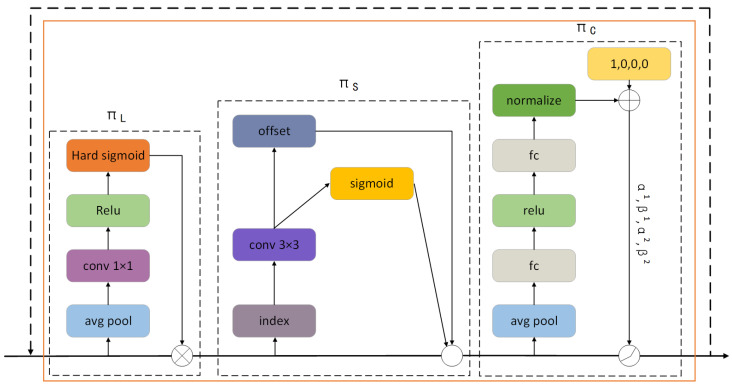
DyHead block structure diagram.

The DyHead detection head can obtain key local features and dynamically assign feature weights, which play an important role in enhancing the robustness and recognition accuracy of the model. The overall network structure diagram is shown in Figure 11, and the structure diagram of a single DyHead Block is shown in Figure 12. Given the feature $F\in R^{L\times S\times C}$ of the input image, L represents the feature level, S is the product of the width and height of the feature map, and C represents the number of channels [25].

(8) $$W\left(F\right)=\pi_{C}\left(\pi_{S}\left(\pi_{L}\left(F\right)\cdot F\right)\cdot F\right)\cdot F$$

where $\pi_{L}$, $\pi_{S}$ and $\pi_{C}$ are the attention functions in the three dimensions L, S, and C, respectively.

The calculation processes of scale-aware attention $\pi_{L}$, spatial-aware attention $\pi_{S}$ and task-aware attention $\pi_{C}$ are shown in the following formulas, respectively.

(9) $$\pi_{L}\left(F\right)\cdot F=\sigma \left(f\left(\frac{1}{S\cdot C}\sum_{S,C}F\right)\right)\cdot F$$

(10) $$\pi_{S}\left(F\right)\cdot F=\frac{1}{L}\sum_{l=1}^{L}\sum_{k=1}^{K}W_{l,k}\cdot F\left(l;p_{k}+△p_{k};c\right)\cdot △m_{k}$$

(11) $$\pi_{C}\left(F\right)\cdot F=max\left(\alpha^{1}\left(F\right)\cdot F_{C}+\beta^{1}\left(F\right),\alpha^{2}\left(F\right)\cdot F_{C}+\beta^{2}\left(F\right)\right)$$

where $\sigma \left(x\right)$ is the hard-sigmoid function, K is the number of sparse sampling positions, $p_{k}+△p_{k}$ is a shifted location by the self-learned spatial offset $△p_{k}$ that focuses on a discriminative region, and $△m_{k}$ is a self-learned importance scalar at the location $p_{k}$.

## 3. Results

### 3.1. Evaluation Indicators

To verify the effectiveness of the proposed cow behavior recognition model DMSF-YOLO, in this study, six evaluation indicators are selected to evaluate the network model. The six evaluation indicators are precision (P), recall (R), mAP50, F1, FPS, parameters, and GFLOPs [27].

The formulas for precision (P), recall (R), mAP, and F1 are shown below.

(12) $$Precision=\frac{TP}{\left(TP+FP\right)}$$

(13) $$Recall=\frac{TP}{\left(TP+FN\right)}$$

(14) $$mAP=\frac{\sum_{i=1}^{C}{AP}_{i}}{C}$$

(15) $$F1=2\cdot \frac{precision\cdot recall}{precision+recall}$$

where TP, FP, and FN, respectively, denote the actual number of positive categories predicted as positive, the actual number of negative categories predicted as positive, and the actual number of positive categories predicted as negative. C is the number of categories, and ${AP}_{i}$ represents the AP value of the i-th category [28].

### 3.2. Experimental Configuration

To avoid the influence of other environmental factors on the training of the model, all experiments in this study are conducted under the same experimental conditions. The experimental configuration of this study is shown in Table 3.

The setting of hyperparameters will also have an important impact on the training of the model. To control the variables, the experiments in this study are also carried out under the same hyperparameters. The hyperparameters imgsz = 640, lr = 0.01 and momentum = 0.937 are based on the default settings of the YOLO series model in Ultralytics, which ensure the stability and accuracy of the model training, and at the same time have a better generalization ability. Batch = 16 is set reasonably according to the GPU memory resources used in this study. The number of samples in each iteration is increased as much as possible within the memory limit, thereby enhancing the stability of training. In terms of optimizer selection, we adopt the fixed pairing of the SGD optimizer that is usually used when lr is 0.01. For epoch = 200, we conducted multiple rounds of experiments under different training rounds to comprehensively evaluate the performance of the training and validation sets, and finally selected this value to ensure that the model learns sufficiently while effectively avoiding underfitting and overfitting problems. The hyperparameter settings are shown in Table 4. Other parameters are the official default parameters of YOLOv11.

### 3.3. Experimental Results

#### 3.3.1. Ablation Experiments

To further explore the contribution of the improved module in the model to overall performance, an ablation experiment was conducted on the test set in the self-built dataset to verify the actual effect. The results are shown in Table 5.

**Figure 13:**
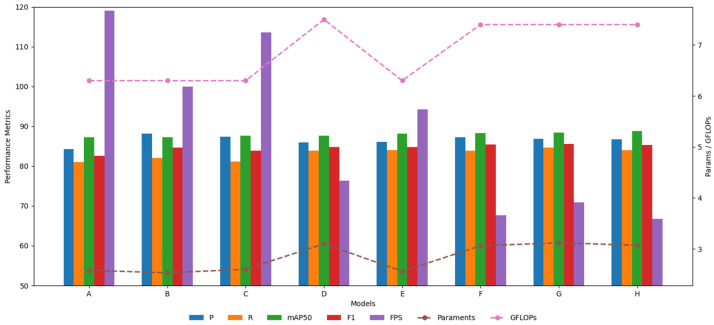
Ablation experiment bar–line graph.

To more intuitively illustrate the effect of the improved model, the data from the ablation experiment are presented in the form of a bar–line graph. Bar graphs and lines of different colors represent different evaluation indicators. Precision (P), recall rate (R), mAP50, F1, and FPS value are represented by bar graphs, and parameters and GFLOPs are represented by line graphs. The horizontal axis represents different models, the vertical axis on the left represents the coordinate scale of the bar graph, and the vertical axis on the right represents the coordinate scale of the line graph. The data effect of the bar graph and line graph is shown in Figure 13.

To more efficiently integrate cow behavior feature information at various scales, the MSFConv module is designed; after replacing this module, the overall precision (P), recall(R), mAP50, and F1 value of model B increased by 3.8%, 1%, 0.1%, and 2%, respectively, compared with the original model A. Considering the background interference of the dairy farm and the detailed information of small multi-scale cows, the C2PSA is innovatively replaced with the designed C2BRA module, and the overall precision (P), recall(R), mAP50, and F1 of model C increased by 3.1%, 0.1%, 0.4%, and 1.3%, respectively. To more effectively extract the multi-scale features of cow behavior and improve the accuracy of cow similar behavior detection, the detection head of the original model is replaced with DyHead. The overall precision (P), recall(R), mAP50 and F1 of the replaced model D increased by 1.7%, 2.8%, 0.4% and 2.2%, respectively. After combining MSFConv and C2BRA for improvement, the overall precision (P), recall (R), mAP50, and F1 of model E increased by 1.8%, 3%, 0.9%, and 2.2%, respectively. Compared with the improvement of a single module, although the precision (P) decreased, the recall (R), mAP50, and F1 values all increased to varying degrees, indicating that the model can reduce the risk of missed detection and has better overall detection performance. Combining MSFConv and Dyhead for improvement, the overall precision (P), recall (R), mAP50, and F1 of model F increased by 2.9%, 2.9%, 1.1%, and 2.8%, respectively. Compared with the improvement of a single module, its precision (P) is lower than that of model B, but significantly higher than that of model D. In addition, the recall (R), mAP50, and F1 values all increased to varying degrees compared with models A and B, indicating that model F captures cow behavior information more comprehensively, effectively improves the comprehensive detection capability, and is more practical for cow multi-behavior category recognition tasks. By combining C2BRA and Dyhead for improvement, the overall precision (P), recall (R), mAP50, and F1 of model G are improved by 2.6%, 3.6%, 1.2%, and 3%, respectively. The precision (P) of model G is lower than that of model C but significantly higher than that of model D. In addition, the recall (R), mAP50, and F1 values are significantly improved, which indicates that model G has stronger behavior capture ability and higher recognition stability in the cow behavior recognition task.

The improved DMSF-YOLO model is 0.6% higher than model E in terms of accuracy, and slightly lower than models F and G. In terms of recall, it is on par with model E, 0.1% higher than model F, and 0.6% lower than model G. In terms of mAP50, it is significantly higher than models E, F, and G. In terms of F1, it is 0.5% higher than model E, and slightly lower than models F and G. Although some indicators of the model DMSF-YOLO after combining MSFConv, C2BRA and Dyhead are slightly lower than those of models E, F and G, they are all at a relatively high level, especially mAP50, which is one of the most core performance indicators and has a greater improvement, indicating that the average detection accuracy of this model is higher. The overall precision (P), recall(R), mAP50 and F1 of the improved DMSF-YOLO model increased by 2.4%, 3%, 1.6% and 2.7%, respectively. Compared with the original model YOLOv11n, although the parameters and GFLOPs of this model have increased slightly, and the FPS has decreased compared with other models, the frame rate still reaches 66.7. While ensuring the improvement of detection accuracy, it also takes into account the higher reasoning speed and has good real-time application potential. This shows that after improving the original model, it can more effectively capture the information of different receptive fields, dynamically focus on key regions and pixels in multiscale feature maps, and significantly enhance its capability in small object detection, enhance the ability of multi-scale feature extraction, and better fuse multi-scale features to improve the accuracy of cow behavior detection.

#### 3.3.2. Comparative Experiments

**Figure 14:**
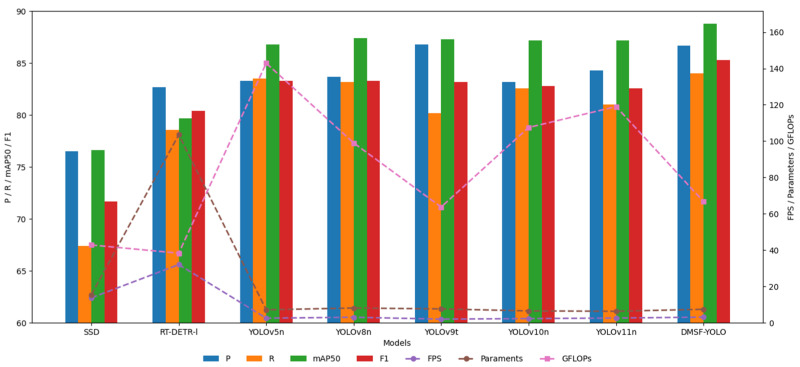
Comparative experiment bar–line graph.

To verify the effectiveness of the improved cow behavior recognition model in this study, the current mainstream target detection models are selected for comparative experiments under the same experimental conditions. As shown in Figure 14, the benchmark model selected in this study is widely used in the field of target detection. With open-source code and better community support, it has good generality. It has been used as an evaluation standard in several cow behavior recognition studies, which can reflect the improvement effect of the model in this study more comprehensively. The results of the comparative experiments are shown in Table 6.

The bar–line graph of the comparative experiment is similar to Figure 13 of the ablation experiment. The horizontal axis of the bar graph of the comparative experiment represents YOLOv5n, YOLOv8n, YOLOv9t, YOLOv10n, YOLOv11n, and DMSF-YOLO models from left to right, and FPS is shown in the form of a line graph.

As shown in Table 6, the precision (P) of the improved DMSF-YOLO model reaches 86.7%, which is 2.4% higher than the original model YOLOv11n and slightly lower than YOLOv9t, but significantly higher than other target detection models. In terms of recall(R), the R-value of the DMSF-YOLO model reaches 84%, which is 3% higher than the original model YOLOv11n and higher than other models, indicating that the model can capture more real positive examples and reduce missed detections. In terms of mAP50, the mAP50 value of the DMSF-YOLO model reaches 88.8%, which is 1.6% higher than the original model and better than other models, indicating that the model can accurately locate and classify different cow behavior categories and has better overall performance. In terms of F1, the F1 value of the DMSF-YOLO model reaches 85.3, which is 2.7% higher than the original model and higher than other target detection models, indicating that the model has better performance. In terms of FPS, the FPS value of the DMSF-YOLO model is lower than that of YOLOv5n, YOLOv8n, YOLOv10n, and YOLOv11n, but significantly lower than that of SSD, RT-DETR-l, and YOLOv9t. Its value reaches 66.7, which fully meets the real-time requirements of practical applications. The parameters and GFLOPs of the DMSF-YOLO model are at an optimal level. The parameters and GFLOPs of the model are slightly higher than those of YOLOv5n, YOLOv8n, YOLOv9t, YOLOv10n, and YOLOv11n but much lower than those of SSD and RT-DETR-l. This shows that the improved model can effectively control the computational and storage costs while maintaining high detection accuracy. In general, the overall performance of the improved cow behavior recognition model DMSF-YOLO is improved to varying degrees compared to the overall performance of the current mainstream target detection model. The model can focus on the key areas of the target cow, extract multi-scale features more effectively, and perform feature fusion. At the same time, it can effectively suppress background interference in the actual cow dairy farm environments and reduce the risk of false detection and missed detection.

#### 3.3.3. Cow Behavior Experiment Analysis

To better validate the model’s effectiveness for cow behavior identification, the mAP50 value of each category is selected as the evaluation index for the experimental analysis, and the cow behavior category detection experimental analysis is carried out on each improved module. The specific results are shown in Table 7.

The model contents indicated by the notes in Table 7 A, B, C, D, E, F, and G are the same as those in the ablation experiment. After replacing C3k2 with the MSFConv module, the mAP50 values of the lying, eating, drinking, and mounting behaviors of model B are all improved compared with the original model. This is due to the excellent multi-scale feature extraction capability of the MSFConv module, which can obtain more feature information. After replacing C2PSA with the designed C2BRA module, the mAP50 values of the lying, standing, walking, and mounting behaviors of model C are all improved compared with the original model. This is due to the beneficial effect that the module automatically obtains information on different scales and performs feature alignment and fusion with the help of a dynamic routing strategy. With the introduction of the DyHead detection head, the mAP50 values of the walking, eating, and mounting behaviors of model D are all improved compared with the original model. This is because DyHead can effectively capture the dynamic behavioral changes of cows, thereby improving the detection ability of the model. After combining MSFConv and Dyhead for improvement, the mAP50 values of model E, except for the mounting behavior, are greatly improved compared with the original model. After combining C2BRA and Dyhead for improvement, the mAP50 values of model F for standing, walking, eating, and drinking behaviors are significantly improved compared with the original model. The mAP50 values of the improved model DMSF-YOLO in the test set for standing, walking, eating, drinking, and mounting behaviors increased by 2.5%, 3.4%, 1.3%, 2.2%, and 0.3%, respectively, with a significant improvement compared to the original model, which indicates that MSFConv, C2BRA and DyHead, can extract information under different receptive fields, extract multi-scale features and perform multi-scale feature fusion more efficiently, reduce the interference of extraneous background factors, improve the ability of small object detection, and effectively distinguish between similar behaviors, and the overall performance of the model is stronger.

#### 3.3.4. Small Target Experiment Analysis

To verify the improvement of the model in small target detection, we set the target whose cow target box accounts for less than 5% of the entire image as a small target, and constructed a small target cow behavior dataset. In the process of constructing the small target dataset, we screen and retain the target boxes whose target box area in the test set is less than 5% of the entire image, and then remove other target boxes, and use the small target cow behavior dataset to verify the model’s detection performance for small targets.

The experimental results of the model before and after the improvement on small targets are shown in Table 8. In terms of precision (P), the improved DMSF-YOLO model improves the accuracy of lying, standing, walking, eating, and drinking behaviors by 0.9%, 5%, 5.1%, 2.1%, and 8.5%, respectively, and the overall accuracy is improved by 3.5%. In terms of recall(R), the improved DMSF-YOLO model increased the recall of lying, standing, walking, eating, and drinking behaviors by 0.5%, 3.4%, 9.6%, 6.3%, and 0.8%, respectively, and the overall recall increased by 3.4%. In terms of mAP50, the improved DMSF-YOLO model improved the mAP50 of standing, walking, eating, drinking, and mounting behaviors by 3.8%, 2.1%, 4.4%, 2.1%, and 4.8%, respectively, and the overall mAP50 by 2.9%. In terms of F1, the improved DMSF-YOLO model improved the F1 values of lying, standing, walking, eating, and drinking behaviors by 0.6%, 4.3%, 7.7%, 4.3%, and 4.1%, respectively, and the overall F1 improvement by 3.2%.

The DMSF-YOLO model’s recognition of cow behaviors has overall different degrees of improvement in each index, and also has different degrees of improvement for different behaviors, which indicates that the improved model DMSF-YOLO has significant improvement in small target detection compared to the original model.

### 3.4. Visual Analytics

#### 3.4.1. Visual Analysis of Model Performance Indicators

**Figure 15:**
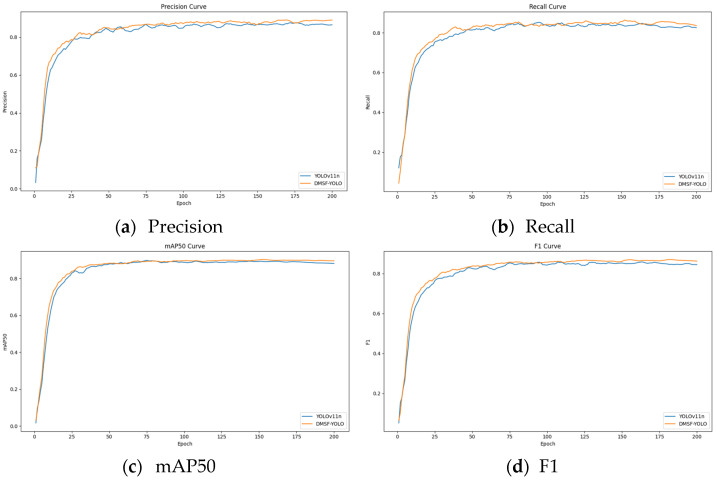
Main performance index curve.

**Figure 16:**
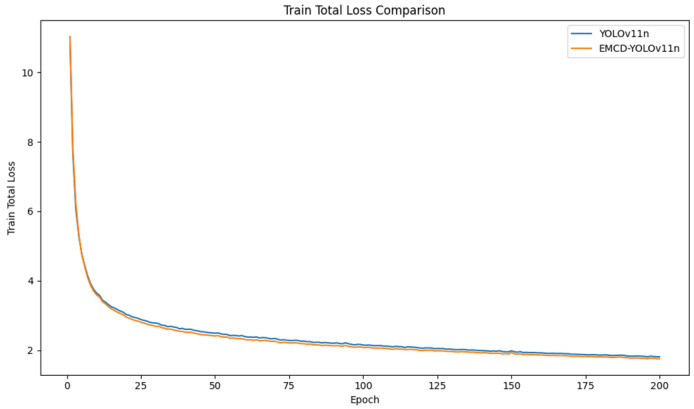
Loss curve comparison curve.

The key performance indicators of the YOLOv11n and improved DMSF-YOLO models during training, including precision (P), recall (R), mAP50, and F1, as well as the overall Loss value, are visualized and analyzed. The curves of the key indicators of the model during training are plotted to observe the dynamic changes of the indicators, as shown in Figure 15 and Figure 16.

By observing the loss curves and comparing the overall loss values before and after the model improvement, the loss curves of the YOLOv11n and DMSF-YOLO models decrease at nearly the same speed; however, after the loss curves stabilize, the loss value of the DMSF-YOLO model is always lower than that of the YOLOv11n. By observing the curves of precision (P), recall (R), mAP50, and F1 indicators, we can see that in the first 10 rounds of training, the indicators increased rapidly. After 50 rounds, the indicators gradually stabilize as the training progresses. The curves of the indicators of the DMSF-YOLO model are smoother than those of the original model YOLOv11n, and all indicators are better than in the original model YOLOv11n. According to the results shown in the figure, while the loss value of the improved DMSF-YOLO model decreases, the performance indicators of the four models are improved to varying degrees, which shows that the overall performance of the improved model is better.

#### 3.4.2. Visual Analysis of Test Results

**Figure 17:**
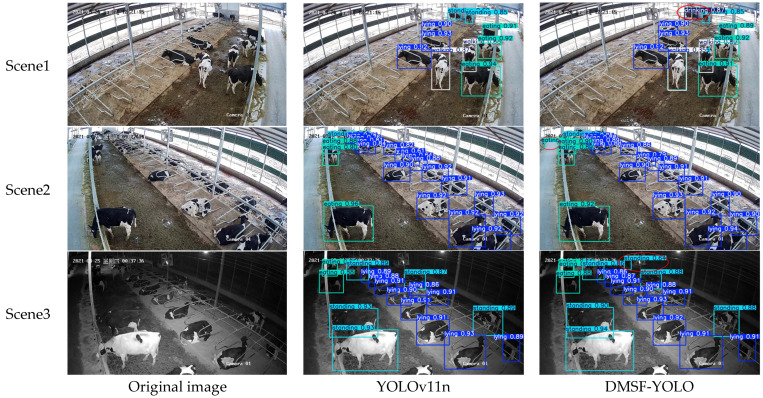
Cow behavior test graph.

To compare the detection effects before and after the model improvement, three representative cow behavior images were extracted from the test set for testing to evaluate the overall performance of the improved DMSF-YOLO model as shown in Figure 17.

Three pictures containing different cow behavior categories are tested and compared with the YOLOv11n model and the DMSF-YOLO model, respectively, and the test results show that the DMSF-YOLO model can accurately recognize cow behaviors. Scenes 1 and 2 are images of cow behavior in daytime environments. By observing the test results in Scene 1, one of the cows’ drinking behaviors is incorrectly identified as standing behavior by YOLOv11n. In contrast, DMSF-YOLO correctly identifies it as drinking behavior, which shows that the model performs well in misdetection. By observing the test results of scene 2, it can be seen that a small target cow eating behavior in the upper left corner of the image is missed by YOLOV11n, while DMSF-YOLO correctly identifies it, which indicates that DMSF-YOLO can detect the small target cows at farther distances under the same scenario, and the recognition ability of the small target is enhanced, which indicates that the model can maintain a high detection level when identifying cows at different scales. Scene 3 is an image of cow behavior under low-light conditions at night. By comparing the test results of the two models, it is found that a cow’s standing behavior in the upper middle position of the image is missed by YOLOV11n, while the DMSF-YOLO identifies it correctly. This indicates that under low-light conditions at night, the DMSF-YOLO model can also maintain a high detection accuracy and improve in missed detection. Secondly, by observing the confidence level of the detection results of the YOLOv11n model and the DMSF-YOLO model in the three scenarios, it can be seen that the confidence level of the improved model has different degrees of improvement relative to that of YOLOv11n, which indicates that the accuracy of the model in recognizing cow behaviors has been significantly improved.

In summary, the improved DMSF-YOLO cow behavior recognition model can detect and identify cows of different scales in complex backgrounds, maintain high detection accuracy in low-light environments at night, enhance the detection and recognition capabilities of small targets, and accurately distinguish different behaviors when facing similar behaviors. The model has been improved in terms of missed detection and false detection, and the overall detection accuracy of the model has also been effectively improved, which can satisfy the needs of cow behavior recognition.

#### 3.4.3. Heatmap Visualization

**Figure 18:**
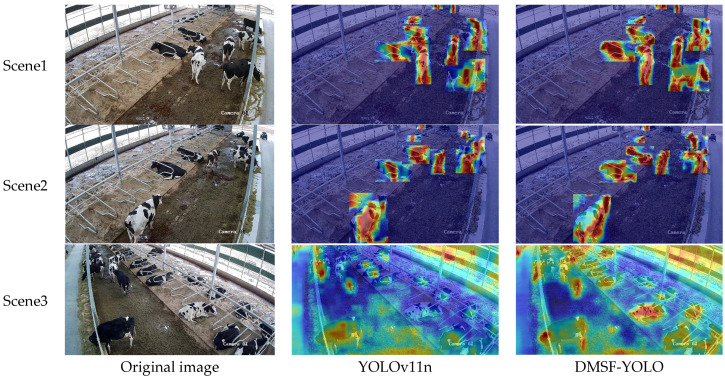
Heat map.

To more intuitively show the effect of the model before and after improvement, GradCAMPlusPlus is used to visualize the key area responses of the original model YOLOv11n, and the improved DMSF-YOLO model. In the heat map, progressive colors are used to indicate the strong and weak distribution of model attention, with blue to red regions representing confidence levels from low to high. Three representative images were selected in the test set for heat map visualization, and the heat map comparison results are shown in Figure 18.

The figure shows the heat map effects of YOLOv11n and DMSF-YOLO in the same scene in three scenes. We only visualize the gradient information in the detection box in scenes 1 and 2, and use a global heat map for visualization in scene 3. Through the comparative analysis of scene 1 and scene 2, we can see that DMSF-YOLO focuses more on the key parts of the cow (such as head and legs) than YOLOv11n, and can show the outline of the cow more clearly. This shows that DMSF-YOLO has a greater capacity to extract key area features, can extract more cow behavior feature information, is conducive to distinguishing similar cow behaviors, and enhances the multi-scale perception ability of the model. Through the comparative analysis of scene 3, we can see that DMSF-YOLO pays more attention to the eating cow in the upper left corner of the picture, and pays less attention to the non-cow area (environmental information). This shows that DMSF-YOLO enhances the detection ability of small targets, can effectively suppress the interference of background information and improves the accuracy of cow behavior recognition.

## 4. Discussion

The rapid and accurate recognition of cow behavior is important for the health management of cows, and the detection results also provide a strong basis for dairy farm management. The DMSF-YOLO model proposed in this study can dynamically extract multi-scale features according to the dynamic mechanism, effectively fuse multi-scale features, reduce the interference of background information, enhance the ability of small target detection, and distinguish similar behaviors by designing the MSFConv module and the C2BRA module and introducing the DyHead detection head. The evaluation indexes of the improved model are all improved to different degrees, and its overall performance is significantly improved compared with the current mainstream target detection model, which has significant advantages in cow behavior recognition.

Although the overall performance of the improved model is better, there are still unresolved issues in the current experimental research. As in this study [19], cow occlusion and different lighting in the cowshed will also pose challenges to the recognition of cow behavior. Therefore, improving the accuracy of cow behavior recognition in extreme scenarios, such as dense cow areas, cows being severely occluded by other objects, and different lighting changes, is also the focus of our next research. In addition, the frequency of small sample cow behavior in farms is low, and it is difficult to capture effective features, which also makes recognition difficult. In the future, in-depth research can be carried out in the directions of multimodal perception, cross-time period modeling, and model generalization ability improvement. The lightweighting of the model is also a key research direction. There is a related study [29] that uses posture estimation algorithms to study the behavior of cows, we can also identify cow behavior from this aspect in the future. In these studies [7,19], lightweight modules are used, or pruning techniques are used to prune the model to reduce the complexity of the model. The number of parameters of the improved model DMSF-YOLO is slightly higher than that of the original model. Future research can focus on the efficiency and real-time performance of the model, reducing the complexity of the model, and achieving lightweight models for easy embedded deployment on mobile terminals.

## 5. Conclusions

In this study, to solve the problems of complex background, multi-scale behavioral changes of cows, similar behaviors, and difficulty in detecting small targets that exist in real dairy farm environments, a cow behavior recognition algorithm DMSF-YOLO based on the fusion of dynamic mechanisms and multi-scale features is proposed, which can accurately identify the behaviors of cows. To better identify the behavior of cows, this study constructed a cow behavior dataset that includes complex backgrounds, multi-scale behavioral changes of cows, similar behaviors, and small target cow individuals, which is used to improve the generalization capacity of the model through data enhancement. To address the multi-scale behavioral changes of cows, the MSFConv module is designed, which extracts global and local features in parallel through four different sizes of convolutional kernels, and combines the channel division mechanism to assign the input features to each scale branch for processing, to achieve efficient multi-scale feature extraction, which can effectively cope with the impact of multi-scale changes of cows, and has significant advantages in multi-scale feature fusion. To address the problems of complex backgrounds, large-scale changes in cows, and difficulty in detecting small targets, the C2BRA module is innovatively designed. After replacing the PSA module in the C2PSA module with the BiLevel Routing Attention (BRA) module, the module automatically selects semantically relevant regions between different scales for attention and interaction with the help of a dynamic routing strategy, aggregates key feature regions of cows, improves the accuracy of feature alignment and fusion efficiency, reduces the interference of irrelevant backgrounds, improves the ability of small target detection, and has significant effects in multi-scale feature fusion. In addition, the dynamic target detection head (DyHead) module was replaced to enhance the model’s multi-scale perception ability, spatial perception ability, and task perception ability, improve the model’s multi-scale target detection performance, focus on the relevant local features of different cow behaviors, and accurately identify different categories of cow behaviors.

The experimental results show that the parameters and GFLOP of the improved cow behavior recognition model DMSF-YOLO are slightly improved compared with the original model, but the overall precision (P), recall (R), mAP50, and F1 of the model are increased by 2.4%, 3%, 1.6%, and 2.7%, respectively. The mAP50 values of the improved model DMSF-YOLO in the test set for standing, walking, eating, drinking, and mounting behaviors increased by 2.5%, 3.4%, 1.3%, 2.2%, and 0.3%, respectively, with a significant improvement compared to the original model. This shows that the DMSF-YOLO model can effectively cope with the impact of multi-scale changes in cows, reduce the interference caused by irrelevant background factors, enhance the ability to recognize small targets, reduce the model’s missed detection and false detection, and accurately classify and identify cow behavior. The overall performance of the DMSF-YOLO model has been significantly improved, which can meet the need for fast and accurate recognition of cow behavior in actual pasture environments and provide a new solution for cow behavior detection.

## Figures and Tables

**Table 1:** Cow behavior determination criteria.

Number	Category	Description of Behavior	Labels	Accounts
①	Lie	Legs bent and body in contact with the ground	lying	13,824
②	Stand	Standing with 4 legs supporting the body and the head at a distance from the ground	standing	11,292
③	Walk	The position of the 4 legs shifted, and the head at a distance from the ground	walking	2652
④	Eat	Standing with 4 legs supporting the body and the head in contact with the ground feed	eating	2886
⑤	Drink	Standing with 4 legs supporting the body and head in contact with the drinking trough	drinking	894
⑥	Mount	More than 1/3 of a cow’s body is crawling across another cow’s body	mounting	276

**Table 2:** Dataset label distribution.

Number	Labels	Train	Val	Test
①	lying	9534	2898	1392
②	standing	7974	2172	1146
③	walking	1908	444	300
④	eating	1974	570	342
⑤	drinking	600	168	126
⑥	mounting	180	66	30

**Table 3:** Environmental configuration.

Configuration Item	Value
Operating System	Windows 10
CPU	Intel^®^ CPU i9-10900K
RAM	64 GB
GPU	NVIDIA GeForce RTX 2080 Ti
Deep Learning Framework	Pytorch 2.2.2 + cu121
CUDAVersion	12.6
Python Version	3.9.21

**Table 4:** Hyperparameter settings.

Parament	Value
Imgsz	640
Batch	16
Lr	0.01
Epoch	200
Optimizer	SGD
Momentum	0.937

**Table 5:** Ablation experiment.

Model	MSFConv	C2BRA	DyHead	P/%	R/%	mAP50/%	F1/%	FPS	Parameters/%	GFLOPs
A				84.3	81	87.2	82.6	119	2.58	6.3
B	√			88.1	82	87.3	84.6	100	2.53	6.3
C		√		87.4	81.1	87.6	83.9	113.6	2.6	6.3
D			√	86	83.8	87.6	84.8	76.3	3.1	7.5
E	√	√		86.1	84	88.1	84.8	94.3	2.56	6.3
F	√		√	87.2	83.9	88.3	85.4	67.6	3.06	7.4
G		√	√	86.9	84.6	88.4	85.6	70.9	3.12	7.4
H	√	√	√	86.7	84	88.8	85.3	66.7	3.07	7.4

Note: A is the original YOLOv11n model; B is the YOLOv11n + MSFConv model; C is the YOLOv11n + C2BRA model; D is the YOLOv11n + Dyhead model; E is the YOLOv11n + MSFConv+C2BRA model; F is the YOLOv11n + MSFConv + Dyhead model; G is the YOLOv11n + C2BRA + Dyhead model; H is the YOLOv11n + MSFConv + C2BRA + Dyhead model.

**Table 6:** Comparative experiment.

Model	Precision (P)/%	Recall(R)/%	mAP50/%	F1/%	FPS	Parameters/10^6^	GFLOPs
SSD	76.5	67.4	76.6	71.7	42.8	13.69	15.4
RT-DETR-l	82.7	78.6	79.7	80.4	38.3	32	103.5
YOLOv5n	83.3	83.5	86.8	83.3	142.9	2.5	7.1
YOLOv8n	83.7	83.2	87.4	83.3	99	3.01	8.1
YOLOv9t	86.8	80.2	87.3	83.2	63.7	1.97	7.6
YOLOv10n	83.2	82.6	87.2	82.8	107.5	2.27	6.5
YOLOv11n	84.3	81	87.2	82.6	119	2.58	6.3
DMSF-YOLO	86.7	84	88.8	85.3	66.7	3.07	7.4

**Table 7:** Cow behavior experiment.

Model	Lying	Standing	Walking	Eating	Drinking	Mounting
A	97.5	84.3	61.1	94.5	86.7	99.2
B	97.6	84	60.2	94.8	87.7	99.5
C	97.8	85.3	62.2	94.2	86.5	99.5
D	97.2	84	63	95.6	86	99.5
E	97.7	84.8	61.8	96	91.1	98.1
F	97.2	85.7	62.7	95.4	90.7	98.4
G	97.3	86.8	64.5	95.8	88.9	99.5

**Table 8:** Small target detection.

Model	YOLOv11n				DMSF-YOLO			
	Precision	Recall	mAP50	F1	Precision	Recall	mAP50	F1
lying	96.7	92.9	97.5	94.8	97.6	93.4	97.4	95.4
standing	68.9	76.9	70.5	72.7	73.9	80.3	74.3	77
walking	59.5	47.7	56	53	64.6	57.3	58.1	60.7
eating	86.1	81.2	84.4	83.6	88.2	87.5	88.8	87.9
drinking	80.3	73	86.7	76.5	88.8	73.8	88.8	80.6
mounting	81.7	94.7	82	87.7	81.5	94.7	86.8	87.6
all	78.9	77.8	79.5	78.3	82.4	81.2	82.4	81.5

## Data Availability

The data presented in this study are available on request from the corresponding author. The data are not publicly available due to an ongoing study.
